# Geographical and temporal trends in imported infections from the tropics requiring inpatient care at the Hospital for Tropical Diseases, London – a 15 year study

**DOI:** 10.1093/trstmh/trw053

**Published:** 2016-09-23

**Authors:** Michael Marks, Margaret Armstrong, Christopher J. M. Whitty, Justin F. Doherty

**Affiliations:** a Hospital for Tropical Diseases, London, UK; b Clinical Research Department, London School of Hygiene and Tropical Medicine, London, UK

**Keywords:** Enteric fever, Febrile illness, Imported infections, Malaria, Travellers

## Abstract

**Background:**

Understanding geographic and temporal trends in imported infections is key to the management of unwell travellers. Many tropical infections can be managed as outpatients, with admission reserved for severe cases.

**Methods:**

We prospectively recorded the diagnosis and travel history of patients admitted between 2000 and 2015. We describe the common tropical and non-tropical infectious diseases and how these varied based on region, reason for travel and over time.

**Results:**

A total of 4362 admissions followed an episode of travel. Falciparum malaria was the most common diagnosis (n=1089). Among individuals who travelled to Africa 1206/1724 (70.0%) had a tropical diagnosis. The risk of a tropical infection was higher among travellers visiting friends and relatives than holidaymakers (OR 2.8, p<0.001). Among travellers to Asia non-tropical infections were more common than tropical infections (349/782, 44.6%), but enteric fever (117, 33.5%) of the tropical infections and dengue (70, 20.1%) remained important. The number of patients admitted with falciparum malaria declined over the study but those of enteric fever and dengue did not.

**Conclusions:**

Most of those arriving from sub-Saharan Africa with an illness requiring admission have a classical tropical infection, and malaria still predominates. In contrast, fewer patients who travelled to Asia have a tropical diagnosis but enteric fever and dengue remain relatively common. Those visiting friends and relatives are most likely to have a tropical infection.

## Introduction

Travel abroad is increasing^1^ with residents of the UK making more than 60 million visits overseas in 2014. Travel to the UK from abroad for holiday, work and immigration has also increased over the last decade. Travel to or from low-income countries is associated with an increased risk of infection, and clinicians will often encounter patients who have become ill either while travelling or shortly after their return. Many illnesses acquired overseas, such as travellers’ diarrhoea, are self-limiting and usually do not present to healthcare providers or can safely be managed in primary care or as outpatients. Some, such as schistosomiasis, are now almost exclusively managed as outpatients.^2,3^ Other imported infections such as malaria or typhoid can be serious or even fatal and patients with these conditions often require admission and specialist input.^4^

Imported infections reflect both current popular travel destinations as well as the movement of members of families which have previously emigrated to the UK and travel to countries where they have friends and family. The spectrum of illnesses seen among returning travellers varies according to both their destination and the incubation period of different infectious agents.^5^ Trends in imported infections are therefore complex and vary according to travel patterns as well as the changing epidemiology of disease transmission overseas. Alongside the classical ‘tropical’ infections, many patients present with illnesses, such as pyelonephritis or respiratory tract infections, that could have been acquired in their country of origin and this must be borne in mind when assessing the returning traveller.

Recently Chikungunya and Zika^6^ have provided examples of how outbreaks can occur outside the disease's historic geographical confines, and increasing drug resistance can lead to spread of bacterial diseases including enteric fevers in areas they were not previously common.^7^ Epidemics of these diseases in the tropics may be associated with increased numbers of cases among travellers. Malaria remains the most common potentially fatal imported infection in the UK and treatment by clinicians experienced in managing imported infections has been shown to be associated with better outcomes.^8^ Although travel to lower income countries is increasing there is, in parallel, widespread development and a major global effort to control or even eliminate classical tropical diseases in many countries so their epidemiology is changing, often rapidly.^9^

We therefore conducted a prospective study to document the spectrum of imported infections sufficiently severe to be admitted to hospital at a major UK specialist tropical referral centre over a 15 year period from August 2000 to February 2015.

## Methods

The Hospital for Tropical Diseases (HTD) is a specialist tropical and infectious disease unit in London including a walk-in service for returned travellers who are unwell. The HTD provides primary, secondary and tertiary care to patients with known or suspected ‘tropical diseases’ throughout the UK; in practice this means those who become unwell on return from low-income countries with tropical climates. In mid-2000 we established a prospective Access database (Microsoft Corp., Redmond, WA, USA) to record data, including demographics, travel and diagnosis, on all admissions to HTD. All admissions where the physician responsible for providing clinical care was a consultant at HTD were recorded in the database. Patients admitted under a different clinical team who only received advice from the HTD team were not included unless care was subsequently transferred to the HTD. A single consultant (JFD) assigned the final diagnosis for all patients one month after discharge based on database information supplemented by review of clinical notes, the discharge letter and all laboratory investigations.

We identified all patients who had travelled and analysed data on demographics, diagnosis, country of travel, reason for travel and duration of stay in hospital. Diseases, such as malaria, for which transmission normally only occurs in tropical regions of the world were classified as ‘tropical diagnoses’. Diseases, such as influenza or urinary tract infection, for which transmission occurs at broadly similar frequency in non-tropical regions were classified as ‘non-tropical’. Individuals who had a self-resolving febrile illness without localising signs and in whom microbiological investigations were negative were considered to have a presumed viral illness. Travel destination was categorised by individual country, UN region and continent. Time since return was categorised as less than 2 weeks, 2 weeks to 3 months or greater than 3 months. In individuals with a ‘non-tropical’ diagnosis we considered travel in the last 12 months to be relevant. For individuals with a tropical diagnosis we considered any travel relevant even if it occurred more than 12 months ago. The reason for travel was categorised as work, holiday, visiting friends and relatives (VFR), immigration, overseas visitors to the UK and military.

### Statistical analyses

Continuous variables were described with mean and SD or with median and IQR as appropriate. Categorical data were described with numbers and percent. Patterns of infection following travel were compared between regions of the world and reasons for travel. Adjusted ORs were calculated controlling for age and sex. All analyses were carried out using Stata 13.1 (StataCorp LP, College Station, TX, USA).

### Ethics approval

The study was reviewed and approved by the Audit and Research Committee at HTD who stated that, as this was a case note study using routine data used for audit, further formal ethical approval was not required.

## Results

Between August 2000 and February 2015 there were 8584 inpatient admissions to HTD made by a total of 7968 patients. Travel history was available for 8349 admissions (93.2%) of which 4362 admissions (51.3%) gave a history of recent travel (within the last 12 months). Travellers were younger than non-travellers (mean 38.0 vs 46.6 years, p<0.0001) and more likely to be male (59.7 vs 54.2%, p<0.0001).

### Travel

Overall, Africa was the most common continent of travel (n=2541) followed by Asia (n=1466) and the Americas (n=690). Most individuals (n=4096/4362, 93.9%) had visited only one country before admission. An accurate estimate of the time between travel and admission was available for approximately half of all admissions (n=1993/4362, 45.7%). The median length of time between returning from travel and admission was 13 days (IQR 6–35) and only 5% of admissions occurred more than one year following travel.

**Figure 1.**
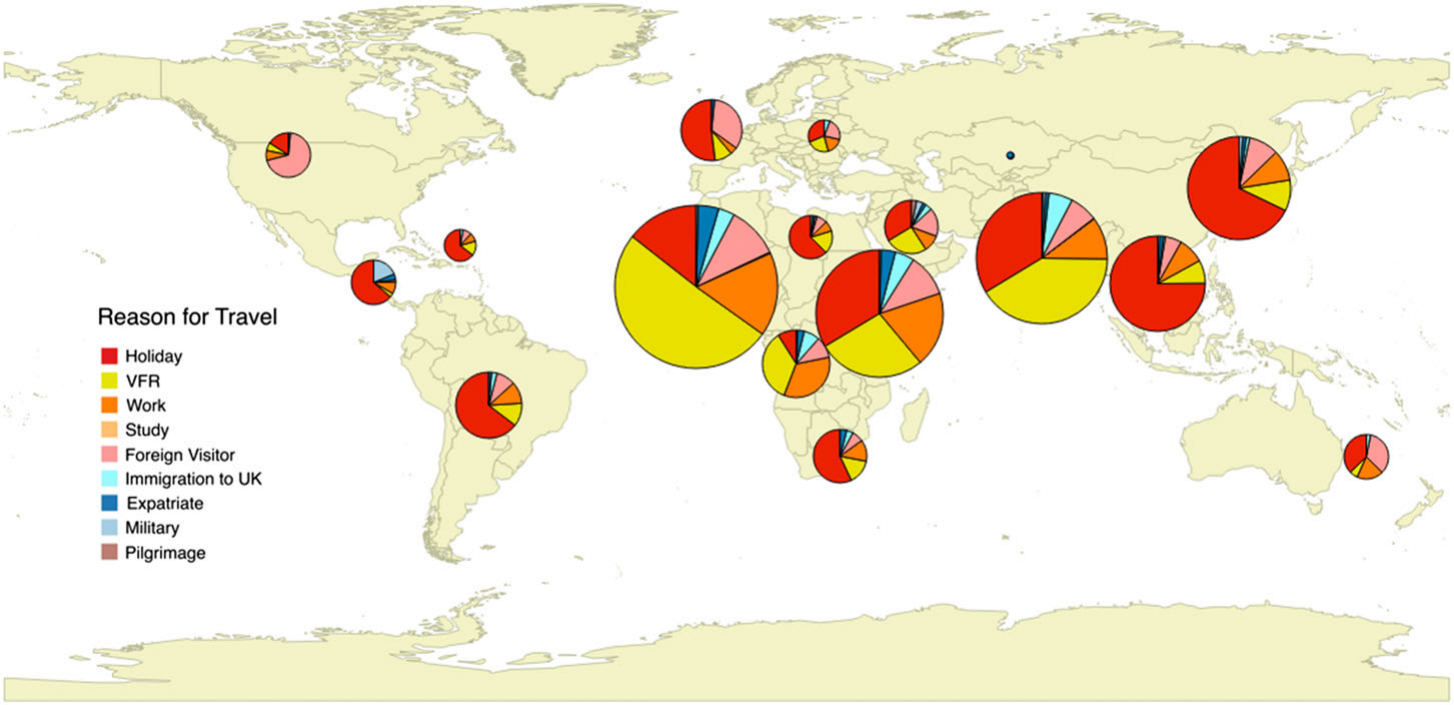
Reason for travel to different regions of the world. The pie charts show the reason for travel in individuals who travelled to each region of the world. Larger pie charts represent regions to which more individuals had travelled. VRF: visiting friends and relatives.

A reason for travel was available for more than three-quarters of admissions (n=3340/4362, 76.6%) (Figure 1 and Table 1). Compared to individuals travelling for holiday, VFR travellers were significantly more likely to be non-Caucasian (93.9 vs 39.7%, p<0.001). Two-thirds of travel by VFRs was to Africa (n=685/1037, 66.1%) compared to just over one-third of travel for tourism (n=413/1143, 36.1%). Among VFR travellers just under half of all travel was to countries in West Africa (n=457/1037, 44.1%). Nigeria, India, and Ghana were the most frequently visited countries (Supplementary Table 1).

**Table 1.** Major reasons for admission following travel by reason for travel

	Holiday	VFR	Work/Study	Expatriate/Foreign visitor	Immigration to the UK	Military	Total
Tropical diagnosis	305	544	174	174	53	20	1270
Falciparum malaria	141	439	122	150	20	3	875
Enteric fever	35	46	18	4	1	3	107
Cutaneous and mucocutaneous leishmaniasis	45	4	6	4	4	12	75
Dengue	43	12	11	4	1	0	71
Non-falciparum malaria	21	15	13	7	3	0	59
Cystic echinococcosis	4	19	0	0	3	1	27
Neurocysticercosis	3	5	2	2	10	0	22
Leprosy	1	3	0	0	10	0	14
African trypanosomiasis	7	0	2	2	0	0	11
Visceral leishmaniasis	5	1	0	1	1	1	9
Non-tropical diagnosis	410	252	154	179	20	3	1018
Presumed viral infection	221	161	105	76	10	2	575
Urinary tract infection	73	39	12	52	3	0	179
Lower respiratory tract infection	56	37	16	28	6	0	143
Skin and soft tissue infection	60	15	21	23	1	1	121
Total	715	796	328	353	73	23	2288

VFR: visiting friends and relatives. A reason for travel was available for a total of 3666 admissions. Data shown here reflect the most common tropical and non-tropical diagnoses made in these individuals.

### Reasons for admission

**Figure 2.**
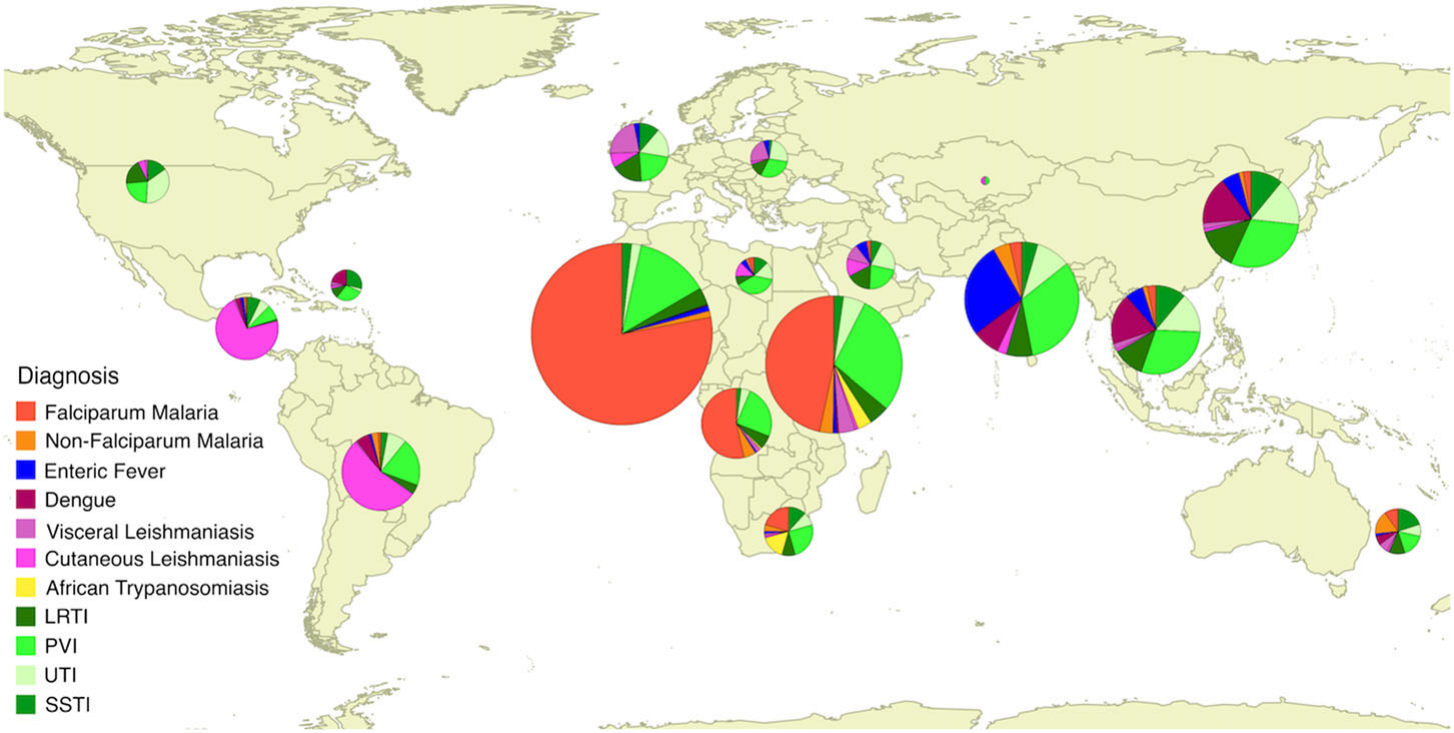
Cause of admission following travel to different regions of the world. The pie charts show the reason of admission in individuals who travelled to each region of the world. Larger pie charts represent regions to which more individuals had travelled. LRTI: lower respiratory tract infection; PVI: presumed viral infection; SSTI: skin and soft tissue infection; UTI: urinary tract infection.

Falciparum malaria was the most common reason for admission (n=1089/4362, 25.0%) (Figure 2 and Table 1). A ‘tropical diagnosis’ was made in just under half of all patients (n=2133, 48.9%) with leishmaniasis (n=242, 4.8%), enteric fever (n=140, 3.1%), dengue (n=98, 2.1%) and non-falciparum malaria (n=78, 1.7%) the most common tropical diagnoses after falciparum malaria. Among individuals who travelled to Africa 1206/1724 (70.0%) had a tropical diagnosis; after malaria, leishmaniasis and human African trypanosomasis were most common, and enteric fever rare (Table 2). Among all travellers to Asia non-tropical infections were less common than tropical infections (349/782, 44.6%) but enteric fever constituting 117 (33.5%) of the tropical infections and dengue 70 (20%) remained important causes (Table 3). Four non-tropical infections were responsible for more than 25% of all admissions: presumed viral illness (n=667, 15.3%), urinary tract infection (n=226, 5.2%), lower respiratory tract infection (n=199, 4.6%) and skin and soft tissue infections (n=144, 3.3%).

**Table 2.** Major reasons for admission following travel to Africa

	Total	Holiday	VFR	Work/Study	Expatriate/Foreign visitor	Immigration to the UK	Military
Tropical diagnosis	1206	160	455	130	151	25	3
Falciparum malaria	1060	133	436	117	143	18	3
Enteric fever	17	5	4	5	2	0	0
Cutaneous and mucocutaneous leishmaniasis	11	2	1	1	0	0	0
Dengue	2	2	0	0	0	0	0
Non-falciparum malaria	38	9	10	5	2	1	0
Cystic echinococcosis	5	1	2	0	0	0	0
Neurocysticercosis	9	0	1	0	2	3	0
Leprosy	11	1	1	0	0	2	0
African trypanosomiasis	28	7	0	2	2	0	0
Visceral leishmaniasis	25	0	0	0	0	1	0
Non-tropical diagnosis	518	123	127	100	48	13	3
Presumed viral infection	342	84	85	73	30	9	2
Urinary tract infection	63	13	17	9	6	1	0
Lower respiratory tract infection	70	10	19	8	10	3	0
Skin and soft tissue infection	43	16	6	10	2	0	1
Total	1724	283	582	230	199	0	6

VFR: visiting friends and relatives. A total of 2160 admissions occurred following travel to Africa. A reason for travel was available for 1741 of these episodes.

**Table 3.** Major reasons for admission following travel to Asia

	Total	Holiday	VFR	Work/Study	Expatriate/Foreign Visitor	Immigration to the UK	Military
Tropical diagnosis	349	86	67	0	17	22	6
Falciparum malaria	22	6	2	3	7	0	0
Enteric fever	117	30	42	14	2	1	3
Cutaneous and mucocutaneous leishmaniasis	21	3	0	0	0	4	2
Dengue	70	34	8	8	2	0	0
Non-falciparum malaria	22	6	5	2	3	1	0
Cystic echinococcosis	30	6	5	2	3	1	0
Neurocysticercosis	15	1	3	1	0	7	0
Leprosy	42	0	2	0	0	8	0
Visceral leishmaniasis	10	0	0	0	0	0	1
Non-tropical diagnosis	433	178	98	0	41	6	1
Presumed viral infection	218	90	61	26	17	0	0
Urinary tract infection	90	41	17	2	10	2	0
Lower respiratory tract infection	76	26	16	3	7	3	0
Skin and soft tissue infection	49	21	4	6	7	1	1
Total	782	264	165	0	58	28	7

VFR: visiting friends and relatives. A total of 1326 admissions occurred after an episode of travel to Asia. A reason for travel was available for 1018 of these episodes.

Tropical infections were significantly more common in VFR travellers compared to holiday makers (n=620/1037, 59.7% vs n=404/1143, 35.4%, p<0.001). Falciparum malaria was the single most common diagnosis among almost all groups of travellers and was the reason for admission in almost half of VFR travellers (n=445/1035, 42.9%). It was also the single most common diagnosis in individuals travelling for work and foreign visitors to the UK. Among individuals travelling for holiday a presumed viral illness was more common than falciparum malaria but this remained the second most common diagnosis overall (n=221/1143, 19.3% vs n=141/1143, 12.3%) and the most common following travel to Africa (n=133, 31.9%) (Table 1).

Among 2279 individuals who had travelled to Africa, falciparum malaria was the single most common diagnosis (n=1068, 46.5%) and was responsible for almost three times as many admissions as the second most common diagnosis, presumed viral illness (n=342, 15.0%). Almost all falciparum malaria occurred following travel to West Africa (n=737/1068, 69.0%) or East Africa (n=252/1068, 23.6%). Overall tropical infections were significantly more common in VFR travellers to Africa compared to holidaymakers (aOR 2.8, 95% CI 2.2–3.6, p<0.0001). This difference was even more marked when comparing the risk of malaria between VFR travellers and holidaymakers (aOR 3.9, 95% CI 2.96–5.02, p<0.0001) (Table 2). Tropical infections were more common than non-tropical infections following travel to West Africa (n=830, 72.2% vs n=295, 20.9%), East Africa (n=350, 44.1% vs n=283, 35.7%), Central Africa (n=116, 55.3% vs n=65, 31.6%) but not following travel to North Africa (n=17, 18.8% vs n=70, 62.5%) and Southern Africa (n=56, 44.8% vs n=56, 44.8%).

Non-tropical diagnoses accounted for almost two thirds of all admissions following travel to Asia (n=618/1317 63.9%). Presumed viral illness (n=218, 16.6%), lower respiratory tract infections (n=78, 5.4%), urinary tract infections (n=90, 6.4%) and skin infections (n=48, 3.5%) were all common. Non-tropical diagnoses were more common in both South Central Asia (n=322, 42.9% vs n=293, 38.5%) and South East Asia (n=198, 53.5% vs n=109, 29.5%). Enteric fever was the most commonly seen tropical diagnosis following travel to Asia overall (n=119, 8.3%) particularly travel to South Central Asia (n=104, 13.3%) and was more common among VFR travellers than holiday makers (n=43/296, 24.3% vs n=31/461, 11.5%, OR 2.6, p<0.001). Dengue was seen in 5.4% (n=71) of travellers to Asia overall but was most common among travellers to South East Asia (n=42/370, 11.4%). Both falciparum malaria (n=22, 1.7%) and non-falciparum malaria (n=22, 1.7%) were rare among travellers to Asia (Table 3).

Helminth infections were seen during a total of 205 admissions (4.7%). A total of 25 individuals were admitted a total of 28 times for treatment of neurocysticercosis. The majority of cases were thought to have been acquired in Asia (n=16, 60%). Thirty six individuals were admitted a total of 58 times with complications of cystic echinococcosis. There were no cases of alveolar echinococcosis. Most were thought to have acquired their infection in the Mediterranean (n=16, 44%) or the Middle East (n=10, 28%). A total of 39 individuals were admitted a total of 45 times with a diagnosis of loaisis which had been acquired in Africa in all cases. Many of the other diagnoses of helminth infections represent likely incidental diagnoses including 29 cases of strongyloidiasis and 10 cases of hookworm. A total of 49 patients with leprosy were admitted over the study (106 admissions), most commonly for complications including skin and soft tissue infections (n=36) and erythema nodosum leprosum (n=20).

**Figure 3.**
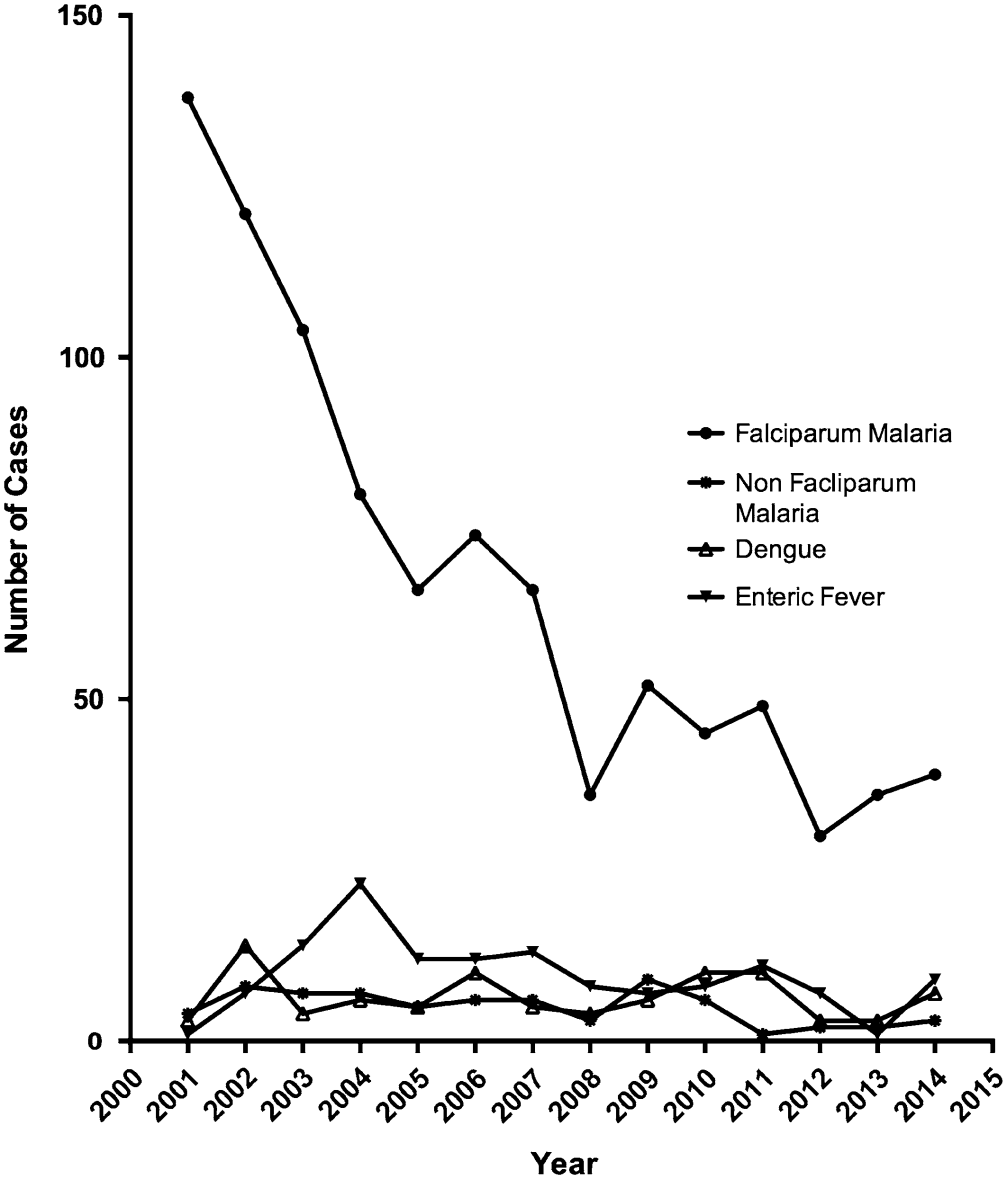
Admissions due to falciparum malaria. The number of admissions per year due to falciparum malaria declined over the period of the study. For comparison the number of admissions related to leishmaniasis, non-falciparum malaria and enteric fever data are also provided. There was no significant change in admission patterns for these three diagnoses.

Over the period of the study the number and proportion of patients admitted with a tropical infection following travel to Africa and Asia declined (Table 4). Although the total number of cases of falciparum malaria in the UK has remained fairly steady,^4^ the number of admissions at our site fell significantly over the study period, from an average of 120 cases per year between 2001 to 2004 to an average of 34 cases a year between 2011 and 2014 (Figure 3). The average duration of hospitalization for cases of falciparum malaria was 3 days (IQR 2–4 days) between 2000 and 2004 and 2 days (IQR 1–3) between 2010 and 2015, although this change was not statistically significant (p=0.49).

**Table 4.** Temporal trends in admissions for tropical infections following travel to Africa and Asia

Diagnosis	Africa			Asia		
	2000–2004	2005–2009	2010–2015	2000–2004	2005–2009	2010–2015
	n=995	n=826	n=454	n=467	n=548	n=300
Falciparum malaria	516 (51.9%)	321 (38.7%)	217 (46.7%)	13 (2.8%)	6 (1.1%)	3 (1.0%)
Non-falciparum malaria	13 (1.3%)	14 (1.7%)	11 (2.4%)	9 (1.9%)	9 (1.6%)	4 (1.3%)
Enteric fever	6 (0.6%)	6 (0.7%)	2 (0.4%)	40 (8.6%)	41 (7.4%)	35 (11.5%)
Dengue	0	2 (0.2%)	0	20 (4.3%)	23 (4.2%)	26 (8.6%)

% are per cent of all admissions following travel to these regions in the time period given.2000–2004 covers the period August 2000 to 31 December 2004; 2005–2009 covers the period 1 January 2005 to 31 December 2009; 2010–2015 covers the period 1 January 2010 to 31 January 2015.

In contrast, there was no change in the number of cases of enteric fever over the course of the study but the average duration of hospitalization declined from 7 days (IQR 4.5–9 days) between 2000 and 2004 to only 4 days (IQR 2–5 days) between 2010 and 2015 (p<0.001). Neither the number of cases of dengue, nor the average duration of hospitalization declined significantly over the course of the study (data not shown).

## Discussion

These data, collected prospectively over 15 years while global travel and epidemiology have changed rapidly, highlight several findings. Firstly, in those recently returned from sub-Saharan Africa classical tropical infections remain the main cause of those severe enough to warrant admission and malaria remains the most important infectious disease requiring admission. Secondly for those who have travelled to other parts of the world outside Africa for most travellers, non-tropical infections, predominantly respiratory, urinary and skin infections are much more common than classical tropical infections, but enteric fever and dengue remain important causes and have not decreased. Thirdly, compared to holidaymakers, individuals travelling to visit friends and relatives remain significantly more likely to acquire a tropical infectious disease regardless of which region of the world they visit. Finally most patients in this study were seen within 2 weeks of return from travel. These findings should inform the triage, investigation and management of unwell returning travellers in the United Kingdom and elsewhere.

Malaria remains the most important infectious disease seen in travellers to Africa. This was particularly true among VFR travellers but was also found among all groups of travellers to Africa, in whom falciparum malaria remained the first or second most common reason for admission regardless of the reason for travel. In contrast both falciparum and non-falciparum malaria were rare in travellers to Asia. We noted a decrease in the number of cases of falciparum malaria seen at HTD over the course of the study, although the total number of cases in the UK has remained fairly constant. This decline might partly reflect changes in epidemiology of falciparum malaria but more importantly changes in patterns of migrant settling of the African diaspora.^10,11^ This decline in patients admitted with malaria may also reflect greater access to specialist care elsewhere and the increased availability of artemisinin based therapies which used to be only available in specialist centres.^12^ Artemisinin based regimes are markedly more effective than older quinine based regimes, particularly in the most unwell patients and wider access to these drugs may have resulted in a reduced need for transfer to a specialist unit.

Unlike malaria, there was no evidence of any decline in the number of cases of enteric fever, especially typhoid. The epidemiology of typhoid, including imported typhoid, is complex and increasingly driven by local patterns of drug resistance.^13^ Rates of dengue diagnosis were unchanged which is likely to reflect the relatively small contribution in London of travellers to Latin America, where there have recently been significant dengue outbreaks, compared to those from Asia.^14^

Marked differences remain between individuals travelling to visit friends and relatives and those travelling for other reasons. More than three-quarters of VFR travellers to Africa had a tropical infection compared to less than half of tourists. A similar, albeit less marked, difference was seen among travellers to Asia. These differences are likely to reflect increased rural travel, longer duration of stay and increased exposure to vectors of disease^4^^,^^15^ and clinicians should consider these differences both when giving pre-travel advice and when investigating individuals returning from abroad.

An important finding is the frequency with which individuals presented with infections that could equally have been acquired in Europe. Clinicians may have a tendency to be biased towards imported causes of infectious diseases in travellers but our data highlight the importance of continuing to investigate for more prosaic infections such as pyelonephritis and respiratory tract infections. These non-tropical infections made up almost two-thirds of infections diagnosed in this study among travellers to Asia and over 30% of infections seen in travellers to Africa.

This study has a number of limitations. Firstly travel data were incomplete or missing in some patients. This included whether individuals had obtained pre-travel advice or appropriate vaccinations. The majority of infections seen in this study were however not vaccine preventable. It is also possible that travel to Europe and North America was not recorded as accurately as travel to Africa and Asia. Whilst this would affect our findings for the spectrum of infections seen following travel to these regions it is unlikely to have had a significant impact on our findings following travel to ‘tropical’ locations. Data from other countries have also reported travel related illness significantly more frequently following travel to Asia and Africa compared to travel to Europe or the Americas.^16^ Secondly we report here only on patients who required admission, who are likely to be at the more severe end of the spectrum. Many illnesses are self-limiting or do not require hospital admission including travellers’ diarrhoea and many arboviral infections. Direct comparison with European surveillance data is not possible as these predominantly report individuals seen in an outpatient setting but in these data^17^^,^^18^ malaria, dengue, influenza-like illnesses and acute gastroenteritis are the most common causes of illness in returning travellers. Given the self-limiting nature of both influenza-like illness and gastroenteritis these illnesses are seen less frequently in patients requiring admission to hospital and this is likely to explain the why these illnesses were seen less frequently in our study. Changes in clinical practice over the course of the study period and an increasing trend for ambulatory management of many tropical infections are also likely to have had an effect on the trends noted in this study. For example we noted a significant decrease in the duration of hospitalization of patients admitted with enteric fever, which might represent an increasing use of azithromycin. Finally, HTD serves as a national referral hospital for tropical medicine so tropical infections may be over-represented compared to the national picture and is likely to explain the relatively high number of patients with chronic infections such as leprosy, cystic echinococcosis and neurocysticercosis treated as inpatients over the course of this study.

Despite these limitations this is a comprehensive study from a single site using a stable methodology looking at the burden of illness following travel in the United Kingdom.

### Conclusions

Our data confirm that falciparum malaria remains the key differential diagnosis following travel to Africa and enteric fever is an important differential from Asia but that non-tropical infections constitute a significant proportion of infectious diseases following travel. Clinicians should bear these differences in mind when managing patients who have returned from travel overseas.

## Supplementary data

Supplementary data are available at Transactions online (http://trstmh.oxfordjournals.org/).

Supplementary Data
