# Supplementary material for: Geographical and temporal trends in imported infections from the tropics requiring inpatient care at the Hospital for Tropical Diseases, London – a 15 year study
Source: Trans R Soc Trop Med Hyg. 2016 Sep 23;110(8):456–63. doi: 10.1093/trstmh/trw053 (PMC5034884; doi:10.1093/trstmh/trw053)
Supplement: Supplementary Data [file supp_110_8_456__index.html]

Geographical and temporal trends in imported infections from the tropics requiring inpatient care at the Hospital for Tropical Diseases, London – a 15 year study — Geographical and temporal trends in imported infections from the tropics requiring inpatient care at the Hospital for Tropical Diseases, London – a 15 year study — Supplementary Data 

# Geographical and temporal trends in imported infections from the tropics requiring inpatient care at the Hospital for Tropical Diseases, London – a 15 year study

## Supplementary Data

Supplementary Data

- Supplementary Data - docx file
