# Supplementary material for: Geographical and temporal trends in imported infections from the tropics requiring inpatient care at the Hospital for Tropical Diseases, London – a 15 year study
Source: Trans R Soc Trop Med Hyg. 2016 Sep 23;110(8):456–63. doi: 10.1093/trstmh/trw053 (PMC5034884; doi:10.1093/trstmh/trw053)
Supplement: Supplementary Data [file supp_trw053_ED_Supp_Table1.docx]

**Supplementary Table 1.** Regions of the world visited by reason for travel

|  | Holiday | Visiting friends or relatives | Work/Study | Expatriate/  Foreign visitor | Immigration to UK | Military | Pilgrimage | Total |
| --- | --- | --- | --- | --- | --- | --- | --- | --- |
| Africa | 426 | 689 | 328 | 248 | 73 | 7 | 0 | 1771  (48.3%) |
| Central Africa | 14 | 55 | 53 | 21 | 12 | 1 | 0 | 156 (4.3%) |
| East Africa | 185 | 151 | 105 | 80 | 27 | 2 | 0 | 550 (15.0%) |
| North Africa | 40 | 11 | 5 | 6 | 1 | 1 | 0 | 64  (1.7%) |
| Southern Africa | 57 | 15 | 13 | 11 | 4 | 0 | 0 | 100  (2.7%) |
| West Africa | 130 | 457 | 152 | 130 | 29 | 3 | 0 | 901  (24.6%) |
| America | 174 | 28 | 30 | 72 | 5 | 13 | 0 | 322  (8.8%) |
| Caribbean | 22 | 5 | 3 | 3 | 1 | 0 | 0 | 34  (0.9%) |
| North America | 11 | 4 | 5 | 49 | 0 | 0 | 0 | 69  (1.9%) |
| Central America | 44 | 2 | 6 | 4 | 0 | 13 | 0 | 69  (1.9%) |
| South America | 97 | 17 | 16 | 16 | 4 | 0 | 0 | 150  (4.1%) |
| Asia | 702 | 321 | 133 | 124 | 44 | 14 | 4 | 1342  (36.6%) |
| Central Asia | 0 | 0 | 0 | 1 | 0 | 0 | 0 | 1  (0.1%) |
| East Asia | 245 | 35 | 35 | 38 | 4 | 4 | 0 | 361  (9.8%) |
| Middle East/ West Asia | 33 | 25 | 10 | 19 | 4 | 4 | 3 | 98  (2.7%) |
| South Central Asia | 194 | 237 | 61 | 45 | 34 | 4 | 1 | 576  (15.7%) |
| South East Asia | 230 | 24 | 27 | 21 | 2 | 2 | 0 | 306  (8.3%) |
| Europe | 78 | 20 | 11 | 53 | 2 | 0 | 0 | 164  (4.5%) |
| Eastern Europe | 11 | 8 | 6 | 8 | 2 | 0 | 0 | 35  (1.0%) |
| Western Europe | 67 | 12 | 5 | 45 | 0 | 0 | 0 | 129  (3.5%) |
| Oceania | 25 | 4 | 13 | 23 | 2 | 0 | 0 | 67  (1.8%) |
| Total | 1405 (38.3%) | 1062 (29.0%) | 515  (14.1%) | 520  (14.2%) | 126  (3.4%) | 34  (0.9%) | 4  (0.1%) | 3666 |
